# Isoform-specific functions of an evolutionarily conserved 3 bp micro-exon alternatively spliced from another exon in *Drosophila homothorax* gene

**DOI:** 10.1038/s41598-020-69644-1

**Published:** 2020-07-30

**Authors:** Ling-Wen Chang, I-Chieh Tseng, Lan-Hsin Wang, Y. Henry Sun

**Affiliations:** 1 Institute of Molecular Biology, Academia Sinica, Taipei, Taiwan, ROC; 2 Institute of Genomic Sciences, National Yang-Ming University, Taipei, Taiwan, ROC; 3 Graduate Institute of Life Sciences, National Defense Medical Center, Taipei, Taiwan; 4 Present Address: Department of Life Science, Chinese Culture University, Taipei, Taiwan, ROC

**Keywords:** Developmental biology, Genetics, Molecular biology

## Abstract

Micro-exons are exons of very small size (usually 3–30 nts). Some micro-exons are alternatively spliced. Their functions, regulation and evolution are largely unknown. Here, we present an example of an alternatively spliced 3 bp micro-exon (micro-Ex8) in the *homothorax* (*hth*) gene in *Drosophila*. Hth is involved in many developmental processes. It contains a MH domain and a TALE-class homeodomain (HD). It binds to another homeodomain Exd via its MH domain to promote the nuclear import of the Hth-Exd complex and serve as a cofactor for Hox proteins. The MH and HD domains in Hth as well as the HTh-Exd interaction are highly conserved in evolution. The alternatively spliced micro-exon lies between the exons encoding the MH and HD domains. We provide clear proof that the micro-Ex8 is produced by alternative splicing from a 48 bp full-length exon 8 (FL-Ex8) and the micro-Ex8 is the first three nt is FL-Ex8. We found that the micro-Ex8 is the ancient form and the 3 + 48 organization of alternatively spliced overlapping exons only emerged in the Schizophora group of Diptera and is absolutely conserved in this group. We then used several strategies to test the in vivo function of the two types of isoforms and found that the micro-Ex8 and FL-Ex8 isoforms have largely overlapping functions but also have non-redundant functions that are tissue-specific, which supports their strong evolutionary conservation. Since the different combinations of protein interaction of Hth with Exd and/or Hox can have different DNA target specificity, our finding of alternatively spliced isoforms adds to the spectrum of structural and functional diversity under developmental regulation.

## Introduction

In eukaryotes, most genes contain exons that are separated by non-coding introns. Mature mRNAs are formed from primary transcripts by excising introns and splicing exons together. Pre-mRNA splicing is conducted by the spliceosome that recognizes the consensus motifs including the 5′ splice site GU, the branch point followed by a short polypyrimidine tract and the 3′ splice site AG^1,2^. The spliceosome is a large ribonucleoprotein complex comprised of five small nuclear ribonucleoproteins (snRNPs, U1, U2, U4/U6, and U5) with small nuclear RNAs (snRNAs) and a number of associated proteins^3^. Alternative splicing confers the flexibility of the pre-mRNA splicing process, and is considered an important trigger for proteome expansion and gene diversity during evolution^4,5^.

Exons of very small size (usually 3–30 nts) are called micro-exons^6,7^. Examples include a 6 bp exon in the *invected* gene in *Drosophila*^8^,a 9 bp exon in wheat *1-SST* gene^9^^,^a 9 bp exon in potato invertase genes^10^,a 1 bp exon in *Arabidopsis APC-11* gene^11^,a 3 bp exon in *Drosophila troponin T* gene^12^,two 5 bp exons in *Drosophila Ubx* gene^6^,two 9 bp exons in *Drosophila Fasciclin 1* gene^13^,an alternatively spliced 3 bp exon in mouse and rat *NCAM* gene^14,15^,a 6 bp exon in chicken cardiac *troponin T* gene 16^16^. Special algorithms were developed to systematically identify the micro-exons, especially from the RNA-Seq data^17–22^. These studies revealed that micro-exons are widely present in eukaryotic genomes^7^. Many of these micro-exons are alternatively spliced and their inclusion or exclusion in transcripts are often highly regulated during development and these regulations are generally conserved in evolution^19,22^.

Alternatively spliced micro-exons, if in-frame, may lead to local changes in protein sequence. If it is not in-frame, it may cause a drastic change of protein sequence or lead to protein truncation. Most micro-exons have a length in multiples of three, therefore are in-frame^19,22^. The local change in protein sequence can affect protein structure, subcellular localization, post-translational modification, enzymatic activity, or protein–protein interactions. Examples are known for effect on protein–protein interaction^19,23^ and post-translational modification^24^. Misregulation of micro-exon alternative splicing may lead to diseases, e.g. autism^25^. Therefore, the identification and functional study of micro-exons in developmentally important genes is vital to the understanding of the full spectrum and diversity of the gene’s functions and regulation.

Homothorax (Hth) is the *Drosophila* homolog of the vertebrate Meis homeodomain (HD) protein family^26–28^. Hth can bind to another homeodomain protein Extradenticle (Exd) to promote the nuclear translocation of Exd, thus making Exd functional in the nucleus by binding to target genes as Exd-Hth complex. The Exd-Hth complex in turn prevents the degradation of the Hth protein^28–31^. The most important feature for Exd-Hth is their function as cofactors of different Hox genes to increase DNA target specificity and contribute to developmental specificity ^32–34^. In addition, Hth-Exd also have Hox-independent functions in development ^28,31,35^.

Hth contains a highly conserved Meis-Hth (MH) domain (also called Homothorax-Meis (HM) domain^26^ and a highly conserved TALE class homeodomain (HD)^26–28^. The N-terminal MH domain interacts with Exd while the C-terminal HD is for DNA-binding to target genes^31,36^. The vertebrate Hth homolog Meis and Prep1 and the Exd homolog Pbx interact similarly^37–40^.

**Figure 1:**
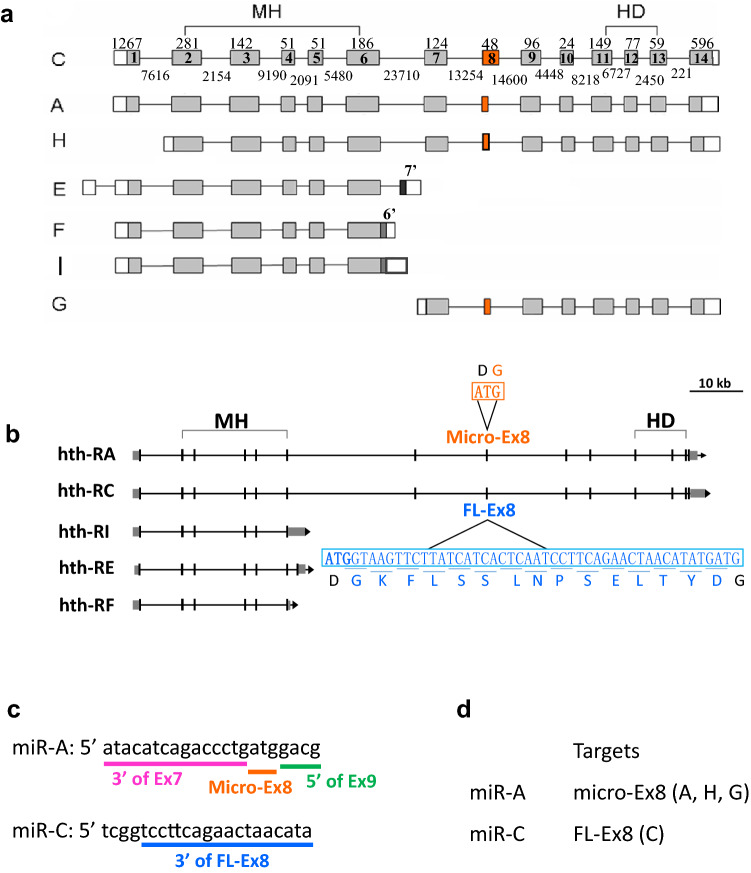
Schematic representation of *hth* isoforms. The *hth* isoforms are depicted. The exons are boxed and numbered. The coding region is in grey. The region of the conserved MH and HD domains are marked. The length (nt) of each exon in Hth-C is marked above the exon, and the length (nt) of each intron in Hth-C is marked below the intron. The length of exon 1 and exon 14 are variable. Hth-C corresponds to clone 7 (GenBank AF035825) in Pai et al., 1998. GenBank AF032865 in Kurant et al., 1998 is similar to isoform C except for shorter exon 1 and exon 14. Hth-A corresponds to clone 5 (GenBank AF036584) in Pai et al., 1998. Hth-H (Rieckhof et al., 1997) lacks exon 1 and has a 5′ extended exon 2, therefore the encoded protein lacks the N-terminal 14 residues, when compared to isoform C. Hth-E and Hth-F corresponds to the isoforms 7′ and 6′, respectively, in Noro et al., 2006. Isoform I is described in FlyBase2020_01. Hth-E is truncated after an alternative exon 7 (7′). Hth-I is truncated after an extended Ex 6. Hth-F is similar to Hth-I, but has a much shorter extended Ex6 (6′). Hth-E, Hth-F and Hth-I each encode a truncated protein with no HD domain. Hth-G (Corsetti and Azpiazu 2013) lacks exons 1–6, therefore can encode a protein without the MH domain. The 48 nt exon 8 (FL-Ex8) and 3 nt exon 8 (micro-Ex8) are marked in red. Interestingly, Hth-A, Hth-H and Hth-G all have the micro-Ex8, while only Hth-C has the FL-Ex8. **b** The Hth-A and Hth-C isoforms are depicted and the sequence for micro-Ex8 and FL-Ex8 are shown. The 3 bp (ATG) of micro-Ex8 corresponds to the first three nt (ATG, in bold) in FL-Ex8. c The sequence of the micro-RNAs for specific knockdown are shown. The correspondence to exons is shown. **d** The design of the isoform-specific miRNAs are shown, with their expected target isoform(s).

The *hth* transcription unit spans 132,008 bp and generates seven isoforms^28,35,41^ (FlyBase 2020_2). These can be grouped into three classes, the long isoforms (Hth-A, Hth-C and Hth-H) that encodes protein containing both MH and HD domains, the MH-only (or HDless) isoform (Hth-E, Hth-F and Hth-I) and the HD-only isoform (Hth-G) (Fig. 1). The Hth-E and Hth-F corresponds to the 7′ and 6′ isoform of Noro et al. (2006)^35^, respectively. The functions of the MH-only and HD-only isoforms have been analyzed^35,41^.

In this study, we report that the *hth* long forms have either a 48 bp exon 8 (FL-Ex8) or a 3 bp micro-exon 8 (micro-Ex8). We provide clear evidence that the micro-Ex8 was generated by alternatively splicing from the FL-Ex8. The micro-Ex8 is actually the first 3 bp in the FL-Ex8, so is an example of alternative splicing of overlapping alternative exons. The functions of these isoforms were examined in vivo by expressing specific isoforms in transgenic flies and by specific knockdown of different isoforms. Interestingly, we found that Hth-A (contains micro-Ex8) and Hth-C (contains FL-Ex8) possess isoform-specific functions although they share similarities in most cellular functions. We showed that the micro-Ex8 is conserved among *Drosophila* species, thereby allowing us to trace the evolutionary history of this alternatively spliced exon to its insect ancestors. The evolutionary conservation provides further support for the functional importance of the alternatively spliced 3 bp micro-Ex8 and the 48 bp FL-Ex8.

## Results

### *hth* isoforms with a 3 bp micro-exon

**Figure 2:**
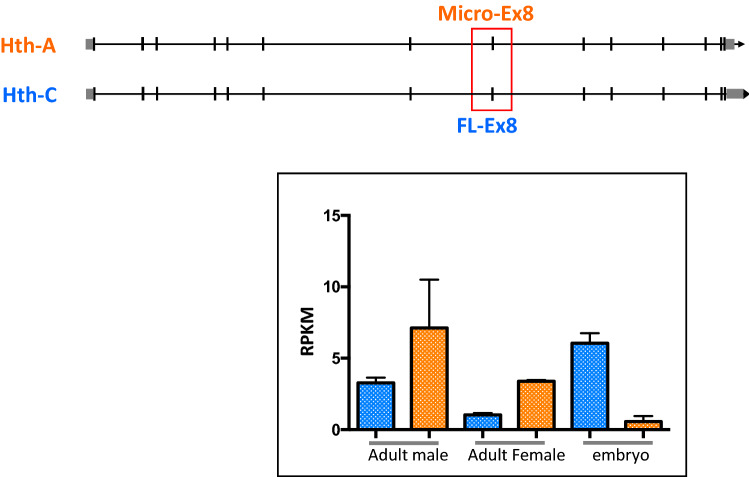
The relative expression of the *hth* micro-Ex8 and FL-Ex8 isoforms. The exon 14 also distinguishes the micro-Ex8 and FL-Ex8 isoforms (see Fig. 1a). Because the micro-Ex8 is too short, we use exon 14 reads to compare their expression level. The relative expression level of micro-Ex8 (orange) and FL-Ex8 (blue) are shown as RPKM (mean ± SEM) mapped to exon 14 of the *hth* gene from adults males or females or hemocytes from stage 16 embryos. Replicates of the raw data have been analyzed. The schematic is the exon–intron organization of *hth-A* and *hth-C*.

The Hth-C isoform has 14 exons and encodes a protein of 487 amino acids with a N-terminal MH domain and a C-terminal HD. It has a 48 bp exon 8 (FL-Ex8). In contrast, the Hth-A, Hth-H and Hth-G isoforms have a 3 bp micro-exon 8 (micro-Ex8). Their encoded proteins differ by 15 amino acids (SDPD**GKFLSSLNPSELTYD**GRW vs. SDPDGRW), which is located in a region between the highly conserved MH and HD domains. UAS-*Hth* constructs have been made using Hth-C^28^ and Hth-H^42,43^. RT-PCR analysis revealed that both isoforms are expressed from all developmental stages (Supplementary Fig. 1). By analyzing RNA-seq data, isoforms containing the FL-Ex8 and micro-Ex8 were expressed in adult males and females and in embryos (Fig. 2). The micro-Ex8 is slightly more abundant than the FL-Ex8 transcripts in adults, but much less abundant than FL-Ex8 in hemocytes from embryos. The HDless isoforms (isoforms E, F and I) are more abundant than the HD-containing isoforms (isoforms A, C, G and H) (Supplementary Fig. 2).

### Micro-Ex8 is alternatively spliced from exon 8

The micro-Ex8 has only three nucleotides and the sequence of these nucleotides is ATG (Fig. 1a). Intriguingly, the first three nucleotide sequence in FL-Ex8 is also ATG, which is flanked by consensus splice acceptor and donor sites (AGATGGT)^44^. These observations suggested that the micro-Ex8 could be generated from the FL-Ex8 through an alternative splicing process. It is possible that these two exons belonged to (1) mutually exclusive exons or (2) usage of alternative 5′ donor sites. To check whether there may be two mutually exclusive exon 8, we addressed three possibilities. The first possibility is that there might be a separate micro-exon located in intron 7 or intron 8. By searching the AGATGGT sequence from exon 7 to exon 9 (including introns 7 and 8), only one such sequence was found in exon 8. We therefore hypothesized that the micro-Ex8 is produced by the AGATGGT sequence located in exon 8.

**Figure 3:**
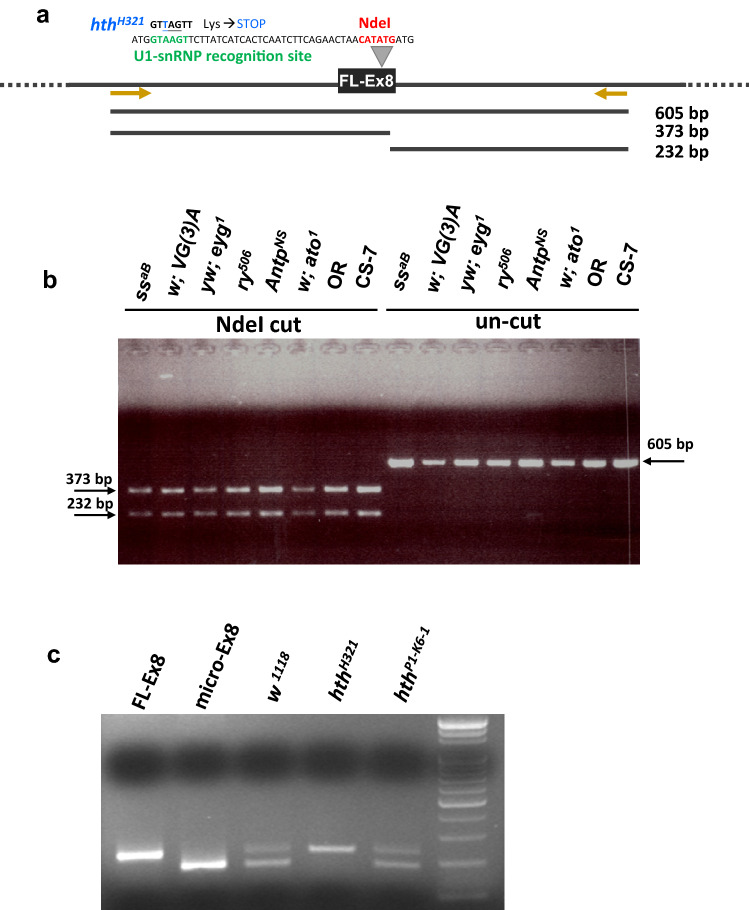
Micro-Ex8 is produced by alternative splicing from FL-Ex8. **a** Schematic representation of FL-Ex8, which contains a potential U1-snRNP recognition site (GTAAGT, in green) and a NdeI cutting site (CATATG, in red). *hth*^*H321*^ harbors a point mutation in exon 8 that changed the U1-snRNP recognition site. The mutation also changed a Lys to a stop codon. The location of primers for PCR are shown as brown arrows and the expected product is 605 bp. Two fragments (373 bp and 232 bp) will be generated after NdeI cutting of the 605 bp PCR product. **b** Six independent homozygous flies and two wild-type strains were tested by PCR of genomic DNA. The PCR products of all strains were 605 bp in length. After NdeI treatment, two fragments of the expected size were generated. **c** By RT-PCR, the *w*^*1118*^ and *hth*^*P1–K6–1*^ produced two bands, corresponding to those produced by in vitro transcription from FL-Ex8 and micro-Ex8 constructs. *hth*^*H321*^ produced a single band, corresponding to the FL-Ex8, indicating that the micro-Ex8 was not produced.

A second possibility is that there might be a genetic polymorphism in exon 8 in the fly population. Although there is a single *hth* gene in the fly genome, it is possible that the two types of isoforms were generated from two *hth* alleles in the fly population. Six fly stocks of independent origins, which are mutants independently isolated by different laboratories, were selected for analysis. Each of these carries a viable mutation on the third chromosome, so the third chromosome was isogenized during mutant isolation. If the two isoforms were from different *hth* alleles, then these fly stocks were expected to each carry only one of the alleles. We checked the six homozygous mutant stocks by PCR of genomic DNA. PCR primers were designed to locate in the introns 7 and 9, thus insuring only genomic DNA can be amplified. A single PCR product of 605 bp was generated from all six fly strains, which contained the full length 48 bp exon 8 (Fig. 3a). The expected product of 540 bp from the putative micro-Ex8 allele was not detected. Since there is a unique NdeI restriction site in the 45 bp stretch that is present in FL-Ex8 but absent in micro-Ex8, only the FL-Ex8 can be digested. Indeed, the PCR products from all six mutant and two wild-type strains, OR and CS-7, contained a NdeI restriction enzyme site and produced the expected products of 373 bp and 232 bp (Fig. 3a,b). The PCR products were also purified from gel and sequenced as a population and confirmed that there was only one type of exon 8 in *hth* in these fly populations (data not shown). These results conclude that micro-Ex8 is not generated by genetic polymorphism in different alleles.

Third, it is possible that polymorphism might exist within the junctions between exon 7/intron 7 or intron 8/exon 9. If this is the case, the 3 bp ATG may be provided by polymorphism at the end of exon 7 or in the beginning of exon 9. PCR primers were designed to amplify the junctions of exon 7/intron 7 and of intron 8/ exon 9. Among the six mutant strains, a single band was detected in the expected size for both PCR products (Supplementary Fig. 3a,b). By sequencing the PCR products, no extra ATG in these junctions was found.

Ruling out the possibilities for the existence of mutually exclusive exons, our results strongly suggest that the micro-Ex8 is generated from FL-Ex8 by alternative RNA splicing. This is further supported by the following analysis of *hth* mutation. *hth*^*H321*^ carries an A to T transition (GTAAGT to GT**T**AGT, Fig. 3a) and changes Lys286 to a stop codon in FL-Ex8^45,46^, thus generating a truncated protein without the HD domain. This same site also constitutes an U1-snRNP recognition site (GTRAGT; R stands for purines)^44^ located immediately downstream of micro-Ex8 (Fig. 3a). The *hth*^*H321*^ mutation changed the U1-snRNP recognition site from GTAAGT to GT**T**AGT. Indeed, no micro-Ex8 isoform was detected by RT-PCR in *hth*^*H321*^ while FL-Ex8 level was elevated (Fig. 3c). This result implies that the production of micro-Ex8 is dependent on the U1-snRNP recognition site.

These results showed that micro-Ex8 is derived from the full length exon8 and its generation requires RNA splicing, thus supporting the idea that micro-Ex8 is generated by alternatively RNA splicing from the FL-Ex8. Here we provide an example of alternative usage of 5′ splice donor sites to choose one exon within another exon. The alternative splicing is in-frame, thus producing two Hth proteins that differ in 15 amino acids stretch located between the two conserved MH and HD domains.

### Evolutionary history of the *hth* micro-exon

**Figure 4:**
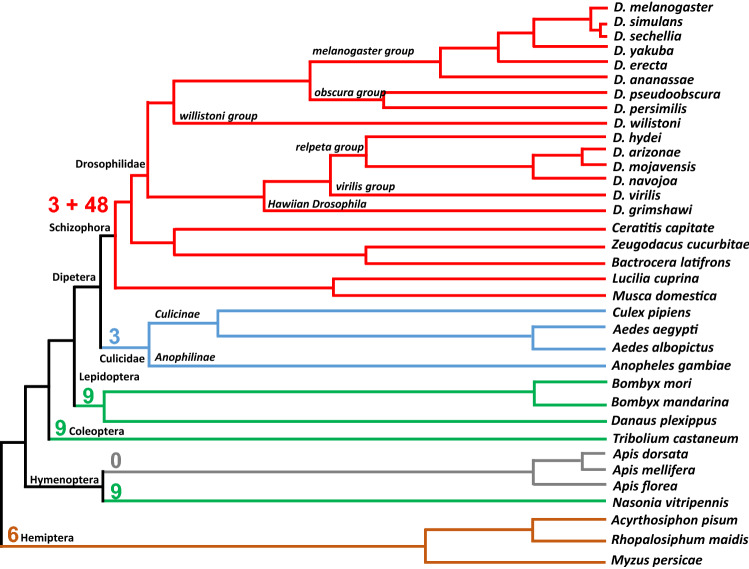
Evolutionary history of the *hth* exon 8. The *hth* exon 8 was analyzed from genomic sequences (from GenBank) of diverse insect species including Diptera, Lepidoptera, Coleoptera, Hymenoptera and Hemiptera. The numbers shown denote the size (bp) of the exon 8. 3 + 48 denote the existence of the 3 bp micro-Ex8 sequence within the 48 bp FL-Ex8.

We then addressed whether these two alternative spliced isoforms exist among insect species. Indeed, this 3 bp micro-Ex8 within a 48 bp FL-Ex8 (designated as 3 + 48) is conserved in all sequenced *Drosophila* species (Fig. 4). The 3 + 48 exon 8 also exist in the house fly *Musca domestica* (Diptera: Muscidae). By using RT-PCR and genomic sequencing to trace the evolutionary origin of this exon, we found that the 3 bp micro-Ex8 (ACG), but not the 48 bp FL-Ex8, exists in *Bombyx mori* and *Aedes aegypti*. *Acyrthosiphon pisum* has a 6 bp exon 8*. Apis mellifera* does not have an exon 8. Based on genomic sequence analysis, another mosquito *Anopheles gambiae* also only has the 3 bp micro-Ex8. On the phylogenetic tree (Fig. 4, Supplementary Fig. 4), we found that the exon began as 6 bp (AATTGG) in the pea aphid *Acyrthosiphon pisum* (Hemiptera)*,* then becomes 9 bp (ACGCCATGG) in the jewel wasp *Nasonia vitripenis* (Hymenoptera), the red flour beetle *Tribolium castaneum* (Coleoptera), the silk worm *Bombyx mori* (Lepidoptera), and then becomes 3 bp (ACG) in the *Culicidae* branch of Diptera which includes the mosquitos *Anopheles gambiae*, *Aedes aegypti* and *Aedes albopictus*. The honeybees (*Apis mellifera*, *Apis dorsata* and *Apis florea*; Hymenoptera) does not have an exon 8. Although the length and coding sequence of exon 8 vary in different insects, the reading frame is preserved in all cases. Altogether, these results suggest that a small exon 8 (9 bp or less) is ancient. The small exon 8 seems to be a constitutive exon. The emergence of the 48 bp FL-Ex8 coincides with the emergence of the Schizophora group ^47^, which include the Drosophilidae and Tephritidae (Fig. 4). The 3/48 bp micro-Ex8/FL-Ex8 combination becomes an alternative splice of overlapping exons.

### Specific 5′ splice site rule for *hth* splicing in insects

Interestingly, we found that the exon boundary of all internal exons in *hth* are defined by the CAG-exon-GTGAGT or CAG-exon-GTAAGT, which is a specific subset of the 5′ splice site motifs^48^. Following this 5′ splice site rule, the annotated exon–intron structure can be re-constituted. For example, the original annotated exon–intron structure of *hth* in *Anopheles gambiae* was based on the CAG-exon-GTGAGG and showed that the Hth proteins are 78% identical in *Aedes aegypti* and *Anopheles gambiae*. After using the 5′ splice site rule to re-constitute the exon boundaries in *hth*, the exon length became better matched between *Aedes aegypti* and *Anopheles gambiae* (Table 1). The Hth protein now shares 91% identity between the two species. Moreover, we found that this rule is applicable for the *hth* gene in Diptera, Lepidoptera, Coleoptera, Hymenoptera and Hemiptera (Table 1).

**Table 1:** The specific 5′ splice site rule for the *hth* genes in insects.

Exon	*Drosophila*		*Aedes*		*Anopheles*		*Bombyx*		*Tribolium*		*Apis*		*Acyrthosiphon*	
	bps	3′ & 5′ splicing recognition site	bps	3′ & 5′ splicing recognition site	bps	3′ & 5′ splicing recognition site	bps	3′ & 5′ splicing recognition site	bps	3′ & 5′ splicing recognition site	bps	3′ & 5′ splicing recognition site	bps	3′ & 5′ splicing recognition site
E2	281	cag–gtgagt	251	cag–gtaagt	254	cag–gtaagt	173	cag–gtgagt	224	cag–gtgagt	260	cag–gtgagt	283	cag–gtaagt
E3	142	cag–gtaaga	142	cag–gtaagt	142	cag–gtaagt	142	cag–gtatgt	142	cag–gtaagc	142	cag–gtaagc	142	tag–gtaata
E4	51	cag–gtaagt	51	cag–gtaagt	51	cag–gtaagt	51	cag–gtaagt	51	cag–gtaagt	51	cag–gtgagt	51	Tag–gtaagg
E5	51	cag–gtaagt	51	cag–gtaagt	51	tag–gtaagt	51	cag–gtgagt	51	cag–gtaagt	51	cag–gtgagt	51	cag–gtaagt
E6	186	cag–gtgagt	186	cag–gtgagt	186	cag–gtgagt	171	cag–gtgagt	195	cag–gtaagt	192	cag–gtgagt	255	cag–gtgagt
E7	124	cag–gtaagt	127	cag–gtatgt	127	cag–gtgagt	130	cag–gtaaga	124	cag–gtaaat	130	cag–gtaagt	130	cag–gtaagt
E8	3 or 48	cag–gtaagt	3	cag–gtaagt	3	cag–gtgagt	9	cag–gtgtgc	9	cag–gtaagt	0	–	6	cag–gtaagt
E9	96	cag–gtaagt	99	cag–gtaagt	99	cag–gtaagt	99	cag–gtaact	108	cag–gtgagt	108	cag–gtaagt	132	cag–gtaagt
E10	24	cag–gtaagc	0	–	0	–	0	–	0	–	0	–	0	–
E11	149	cag–gtgagt	149	cag–gtgagt	149	cag–gtaagt	152	cag–gtaaga	149	cag–gtaagt	149	cag–gtaagt	152	tag–gtaagc
E12	77	cag–gtgagt	77	cag–gtgagt	77	cag–gtaagt	77	cag–gtaata	77	cag–gtgagt	77	cag–gtaagt	77	cag–gtaagt
E13	59	cag–gtaact	59	cag–gtaata	59	cag–gttagt	59	cag–gtgtgt	59	cag–gtaagt	59	cag–gtaaga	59	cag–gtaagt

The 3′ and 5′ splice recognition sites for the internal exons in the *hth* gene from diverse insect species: *Drosophila melanogaster* (Diptera: Drosophilidae), *Aedes aegypti* (Diptera: Culicidae), *Anopheles gambiae* (Diptera: Culicidae), *Bombyx mori* (Lepidoptera), *Tribolium castaneum* (Coleoptera), *Apis mellifera* (Hymenoptera) and *Acyrthosiphon pisum* (Hemiptera). The length of each exon is also listed. The exon boundary of the internal exons in *hth* can be defined by CAG-exon-GTGAGT or CAG-exon-GTAAGT, which is a specific subset of the 5′ splice site motifs (Yeo and Burge, 2004). The length of exons 3, 4, 5, 12 and 13 are conserved. The other exons vary slightly in length, but the variations are always in-frame. The 24 bp exon 10 of *hth* is absent in all of the tested non-*Drosophila* species.

### Isoform-specific knockdown by microRNAs

To study the functions of distinct Hth isoforms, we generated UAS transgenes of microRNAs targeting to different regions of the *hth* transcripts (Fig. 1c,d). miR-C targets the 3′ portion of FL-Ex8 and is thus expected to specifically knockdown the FL-Ex8 isoform (Hth-C). miR-A targets the junction in Ex7/micro-Ex8/Ex9, so is expected to specifically knockdown the micro-Ex8 isoforms (Hth-A, Hth-H and Hth-G).

**Figure 5:**
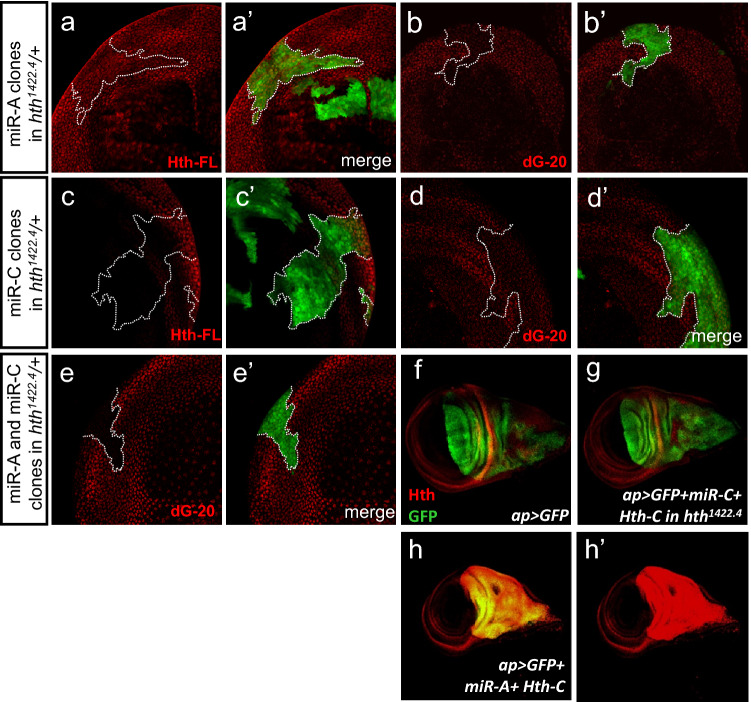
Specific knockdown of Hth-A or Hth-C by miRNA in the imaginal discs. Clonal expression of miR-A (**a**–**b**′) or miR-C (**c**–**d**′) in *hth*^*1422–4*^*/* + heterozygotes. The clones are marked by GFP (green). The effect on Hth (red) level is measured by dG-20 that specifically recognizes the C-terminal of Hth (**b**, **b**′, **d**, **d**′) and anti-Hth-FL (**a**, **a**′, **c**, **c**′). The anti-Hth-FL detected no obvious reduction in clones expressing miR-A (**a**, **a**′) or miR-C (**c**, **c**′). The dG-20 signal was significantly reduced in clones expressing miR-A (**b**) and miR-C (**d**). When miR-A and miR-C were coexpressed, the dG-20 signal was eliminated (**e**, **e**′). (**f**–**h**′) Hth level (detected by dG-20; red) in the wing discs of **f** *ap* > *GFP*, **g** *ap* > *GFP* + *miR-C* + *Hth-C* in *hth*^*1422–4*^*/* + heterozygotes, and **h** *ap* > *GFP* + *miR-A* + *Hth-C*. GFP (green) shows the *ap-GAL4* expression pattern. Note that ectopic expression of Hth-C was not attenuated by co-expression of miR-A (**h**′) but is completely knocked down by miR-C (**g**).

We generated clones that express each of these microRNAs in imaginal discs of *hth*^*1422–4*^*/* + and used anti-Hth antisera to determine the effects by knockdown of distinct Hth isoforms. An antiserum (dG-20)^49,50^ generated against the C-terminal part of Hth, cannot detect the three HDless isoforms^35^. Clonal expression of miR-A significantly reduced the dG-20 signal (Fig. 5b,b′). Similarly, clonal expression of miR-C significantly reduced the dG-20 signal (Fig. 5d,d′). When miR-A and miR-C were coexpressed, the dG-20 signal was eliminated (Fig. 5e,e′). These results indicated that the microRNA knockdown was efficient, at least in an *hth*^*1422–4*^*/* + background when the *hth* gene dosage is reduced. The results suggest that both Hth-A, Hth-C and Hth-G contribute to adequate level of Hth, which is also supported by our previous findings (Fig. 2 and Supplementary Fig. 1).

An anti-HthFL antiserum, generated against the full length Hth^28^^,^ is expected to detect all Hth isoforms. In clones that express miR-A or miR-C alone, the anti-HthFL signal was only slightly reduced (Fig. 5a,a′,c,c′). When miR-A and miR-C were coexpressed, the anti-HthFL signal was not significantly reduced (data not shown). This result suggest that the long isoforms (Hth-A, Hth-H and Hth-C) constitute only a minor proportion of the endogenous Hth, consistent with the previous finding that the HDless isoforms make up a major proportion of the total Hth level^35^. This is also supported by RNA-seq data (Supplementary Fig. 2).

The miR-A sequence contains 19 nucleotides in the exon 7, raising the possibility that it may target to all isoforms with exon 7 (Hth-A, Hth-C, Hth-H and Hth-G) (Fig. 1a). To better understand the knockdown specificity of miR-A and miR-C, we combined ectopic expression and knockdown. We used the *ap-Gal4* to ectopically express Hth-C (abbreviated as *ap* > *Hth-C*) in the dorsal region of the wing disc, which is largely non-overlapping with the endogenous Hth in the outer region of the prospective wing pouch (Fig. 5f, visualized by expressing GFP). In *ap* > *miR-A* + *Hth-C*, the Hth level was elevated significantly in the *ap* expression domain (Fig. 5h–h’, compare with f), indicating that the miR-A could not knockdown Hth-C. In *ap* > *miR-C* + *Hth-C*, the Hth was not detected in the *ap* expression domain (Fig. 5g, compare with h), except where the *hth* and *ap* expression domains overlap. In this overlap region, the Hth level was slightly reduced (Fig. 5g, compare with f). These results indicate that the miR-C can efficiently knockdown Hth-C. These results indicate that the FL-Ex8 (represented by Hth-C) can be efficiently knocked down by miR-C but not by miR-A, and also ruled out the possibility that miR-A might affect both Hth-A and Hth-C. This result is consistent with the conclusion that the sequence at 5′ end of micro RNA is less important for its RNAi function^51^.

### Functional differences between isoforms containing FL-Ex8 and micro-Ex8

We asked whether the micro-Ex8 and FL-Ex8 isoforms may have distinct functions in vivo. First, we compared the effects of overexpressing the different isoforms. When Hth-A or Hth-C were ectopically expressed outside of the endogenous *hth* expression pattern (driven by *ey-Gal4, ap-Gal4, dpp-Gal4, en-Gal4, and Dll-Gal4*), both isoforms gave similar phenotypes. *ey* > *Hth-C* and *ey* > *Hth-A* similarly caused reduction or absence of eyes. *Dll* > *Hth-C* and *Dll* > *Hth-A* similarly caused absence of antennal a3 segment, truncated legs with no tarsus and tibia, and shorter palp. *ap* > *Hth-C* and *ap* > *Hth-A* similarly caused significant pupal lethality. The escapers have smaller notum and scutellum, less bristles and smaller and blistered wings (Fig. 7g,h). *dpp* > *Hth-C* and *dpp* > *Hth-A* similarly caused absence of eye, arista and palp, less bristles on the notum, truncated legs with no distal segments or no tibia but with tarsal segments. *en* > *Hth-C* and *en* > *Hth-A* similarly caused shorter femur, tibia and tarsus. Therefore, ectopic expression of Hth isoforms outside of the *hth* expression domain does not reveal functional differences between the FL-Ex8 and micro-Ex8 isoforms.

**Figure 6:**
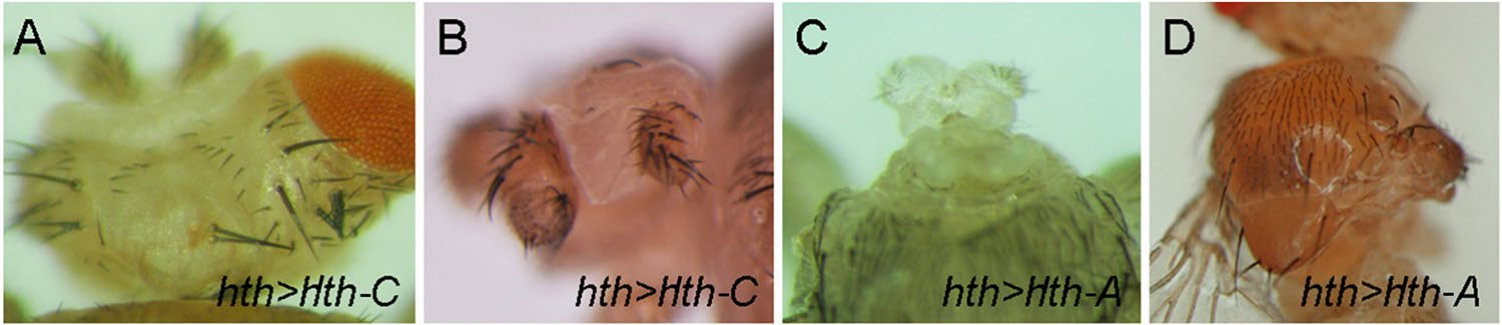
miRNA target specificity on isoforms with FL-EX8 and micro-EX8. **a**–**d** Overexpression of Hth-C (**a**, **b**) and Hth-A (**c**, **d**) in the *hth* endogenous expression pattern by *Hth-GAL4*. **a**, **b** *hth* > *Hth-C* caused pupal lethality. The escapers have a reduction or absence of eyes; deformed ocellar region; deformed or absence of palps; antenna with shorter a3 segment; some with ectopic antennal a2 segment in eye region; some with no or only one haltere; and disordered vibrissae. **c**–**d** *hth* > *Hth-A* caused phenotypes similar to *hth* > *Hth-C*. In addition, some flies are headless (**c**) or wingless (**d**).

However, when Hth-A or Hth-C were overexpressed in the endogenous *hth* expression domain (*hth* > *Hth-A* and *hth* > *Hth-C*), some different phenotypes could be detected. Both genotypes caused significant pupal lethality (Table 2). The *hth* > *Hth-A* and *hth* > *Hth-C* escapers showed similar defects in multiple organs, such as the reduction or absence of eyes; deformed ocellar region; deformed or absence of palps; antenna with shorter a3 segment; some with ectopic antennal a2 segment in eye region; some with no or only one haltere; and disordered vibrissae (Fig. 6a,b). The only difference is that *hth* > *Hth-A* caused headless or wingless phenotypes with 12% and 6% penetrance, respectively (Fig. 6c,d), while *hth* > *Hth-C* does not. Since all the other phenotypes were similar, these differences cannot be attributed to a difference in protein expression level. The headless and wingless phenotypes caused by *hth* > *Hth* flies (*UAS-GFP-Hth*; Hth-H)^43^ have been described^35^. Therefore, the micro-Ex8 isoforms (Hth-A and Hth-H) have overexpression effect on head and wing development which is not induced by overexpression of the FL-Ex8 Hth-C.

**Table 2:** Phenotypes of overexpression and/or knockdown of specific isoform.

Genotype	Phenotypes	Pupal lethality %
*hth* > *Hth-C*	Reduction or absence of eyes; deformed ocellar region; deformed or absence of palps; antenna with shorter a3 segment; some with ectopic antennal a2 segment in eye region; some with no or only one haltere; and disordered vibrissae	78% (n = 49)
*hth* > *Hth-A*	Similar to *hth* > *Hth-C*. Headless (12%) or wingless (6%)	87% (n = 54)
*hth* > *miR-A*	Normal head capsule and clypeus. No antenna-to-leg transformation	100% (n = 154)
*hth* > *miR-C*	Normal head capsule and clypeus. No antenna-to-leg transformation	100% (n = 125)
*hth* > *miR-A* + *Hth-A*	58% fully rescued. Some with deformed eyes and palps. Some with shorter antennae	6% (n = 135)
*hth* > *miR-A* + *Hth-C*	66% fully rescued. Pharate adults have deformed palps; shorter antennae	5% (n = 101)
*hth* > *miR-C* + *Hth-A* in *hth1422-1/* +	Small or no eyes; disordered vibrissae; no or reduced ocelli; no or deformed palps; shorter antennal a3 segment. 31% pupae without head	98% (n = 67)
*hth* > *miR-C* + *Hth-C* in *hth1422.4/* +	Shorter antennae, deformed or no palps and disordered vibrissae	58% (n = 33)

The phenotypes of overexpressing isoforms Hth-A and Hth-C and specific knockdown by miR-A and miR-C are described. The phenotypes of coexpressing different miR with Hth-A or Hth-C are also described.

Secondly, we examined the effect of specific knockdown of each isoform using *hth-GAL4* (Table 2). *hth* > *miR-A* and *hth* > *miR-C* caused 100% pupal lethality but has normal head capsule and clypeus and no antenna-to-leg transformation. Reducing *hth* dosage in *hth*^*1422–1*^*/* + did not enhance the phenotype. Since knockdown of either FL-Ex8 or micro-Ex8 isoforms caused pupal lethality, it suggests that the two isoforms have non-redundant functions and both are required. Knockdown of both FL-Ex8 and micro-Ex8 isoforms caused developmental defects not induced by single knockdown, suggesting that for many developmental processes, the two types of isoforms have redundant functions and both have to be knocked down to cause the developmental defects.

Thirdly, we checked whether the knockdown of one type of isoform can be functionally substituted by overexpressing another type (Table 2). We combined microRNA knockdown and overexpression, using the same *hth-GAL4*. In many cases we added *UAS-GFP* to equalize the number of *UAS* constructs. Coexpression of GFP did not alter the phenotypes. Knockdown by miR-A or miR-C all caused 100% pupal lethality. In *hth* > *miR-A*, coexpressing Hth-A or Hth-C gave nearly complete rescue of the pupal lethality and significant rescue of the developmental defects. This suggests that Hth-C (FL-Ex8) can functionally replace Hth-A (micro-Ex8) to a large extent. In *hth* > *miR-C*, coexpressing Hth-C gave significant rescue of the pupal lethality (58%) and significant rescue of the developmental defects. However, coexpressing Hth-A failed to rescue (98% lethality) and the pharates have phenotypes similar to those of *hth* > *Hth-A*. The lack of rescue indicates that Hth-A (micro-Ex8) cannot functionally replace the loss of Hth-C (FL-Ex8). These results suggest a functional difference between isoforms with FL-Ex8 or micro-Ex8. This difference is unlikely due to difference in expression level nor affected by the number of *UAS* constructs.

**Figure 7:**
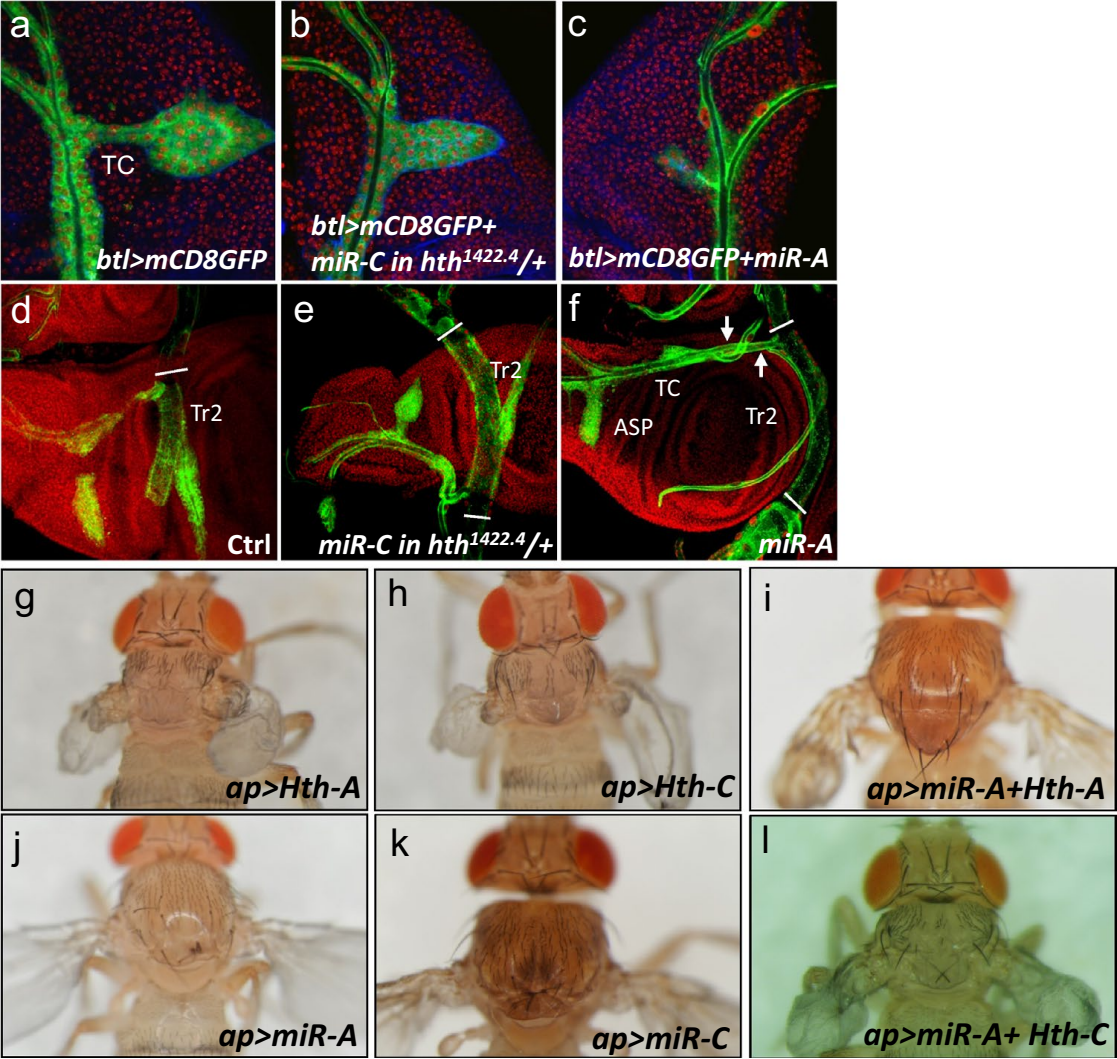
Isoforms A and C can have distinct effects upon overexpression. **a**–**f** Tracheal development in late third instar wing disc is visualized by *btl* > *mCD8-GFP* (green). Knockdown of Hth-C in trachea in *btl* > *miR-C* + *mCD8-GFP* in *hth*^*1422–1*^*/* +) did not affect the cell repopulation of Tr2 and TC (**b**, **e** compared with **a**, **d**). In contrast, expression of miR-A (*btl* > *miR-A* + *mCD8-GFP* in *hth*^*1422–1*^*/* +) caused a delay of cell repopulation in trachea (**c**, **f**) and only 45% of adults eclosed (compared to 96% in *btl* > *miR-C* in *hth*^*1422–4*^*/* +). Tr2, tracheal metamere 2; ASP, air sac primordium; TC, transverse connective. **g**–**l** Adult thorax phenotypes of **g** *ap* > *Hth-A*, **h** *ap* > *Hth-C*, **i** *ap* > *mir-A* + *Hth-A*, **j** *ap* > *miR-A*, **k** *ap* > *miR-C* and **l** *ap* > *miR-A* + *Hth-C*. Note that phenotype caused by ectopic expression of Hth-A (**g**) was rescued by co-expression of miR-A (**i**). But the *ap* > *Hth-C* phenotypes (**h**) was not rescued by coexpressing miR-A (**l**).

*ap* > *Hth-A* and *ap* > *Hth-C* caused similar developmental defects (Fig. 7g,h). As expected, the *ap* > *Hth-A* overexpression phenotype could be rescued by coexpressing miR-A (*ap* > *miR-A* + *Hth-A*; Fig. 7i), and the *ap* > *Hth-C* overexpression phenotype could be rescued by coexpressing miR-C (*ap* > *miR-C* + *Hth-C*; data not shown). Significantly, the *ap* > *Hth-C* phenotypes could not be rescued by coexpressing miR-A (*ap* > *miR-A* + *Hth-C*) (Fig. 7j,k,l).

Hth is also involved in tracheal development. At the end of larval stage, the large larval cells in the tracheal metamere Tr2 can divide and become small imaginal cells and incorporate into the adult tracheal system and this repopulation requires *hth*^52^. We checked which isoforms is required for tracheal development. Hth-A or Hth-C was knocked down in the tracheal cells by the *btl-GAL4* in a *hth*^*1422–4*^*/* + heterozygous background (Fig. 7a–f). Tracheal development was not affected in *btl* > *miR-C* flies (Fig. 7b,e) while *btl* > *miR-A* induced a delay of cell repopulation of tracheae (Fig. 7c,f, compare with Fig. 7a,d). Notably, 96% of *btl* > *miR-C* pharate adult has eclosed from the pupa while only 45% of *btl* > *miR-A* has eclosed. Taken together, these results imply that Hth-A plays a primary role and Hth-C might play a minor role in the repopulation in Tr2 and TC.

## Discussion

### A 3 bp micro-exon generated by alternative splicing in the *hth* gene

In this study, we have provided unequivocal evidence that the Hth-A and Hth-C isoforms of *hth* differ by the choice of micro-Ex8 or FL-Ex8 through alternative splicing. We first showed that both isoforms are actually expressed, evident by RT-PCR and RNA-Seq. We then ruled out possibilities other than alternative splicing that may explain the existence of two isoforms. Finally, we showed that alteration in the U1-snRNP recognition site located immediately downstream of micro-Ex8 completely abolished the production of micro-Ex8. Therefore, micro-Ex8 and FL-Ex8 are produced by alternative splicing. This is an interesting example of alternative choice of overlapping exons.

Previous studies showed that the short-length of micro-exons are difficult to be recognized by the splicing machinery and can cause exon skipping^53^. More recently, it has been reported that micro-exons have stronger 5′ and 3′ splice sites and higher density of splicing enhancers. These may promote the association of RNA binding proteins and cause inclusion or exclusion of alternative exons^22^. The inclusion or exclusion of alternative exons can affect protein function such as altering protein structure, modulating post-translational modification or facilitating degradation^54,55^ and may contribute to human disease. In the Hth case reported here, the alternative 5′ splice site choice of Hth results in a 15 aa deletion (from FL-Ex8 to micro-Ex8) in the Hth protein. This is similar to the human Wilms’ tumor suppressor gene WT, which has an alternatively spliced exon 9, resulting in the inclusion or exclusion of three amino acids (KTS)^56^. Mice expressing only + KTS or only − KTS splice isoforms have distinct and severe phenotypes in kidney and gonad formation^57^^,^ indicating these two isoforms have differential functions on urogenital development. Mutations affecting the alternative slicing results in the Frasier syndrome with gonadal dysgenesis and kidney dysfunction^58^. Hence, micro-exons are attractive model for studying alternative splicing to aid the understanding of biological functions and processes.

### Evolutionary history of the micro-exon 8

Our analysis of available genomic sequences showed that a small and constitutive exon 8 (9 bp or less) in the *hth* gene is ancient. It appeared in the holometabolous insects. Although the length and coding sequence of exon 8 vary in different insects (0, 3, 6, 9), the reading frame is preserved in all cases. The emergence of the 48 bp FL-Ex8 in the Schizophora group, which include the Drosophilidae and Tephritidae (Fig. 4), suggests it is more derived feature.

The isoforms A and C differ by 15 amino acids (GKFLSSLNPSELTYD) located between the conserved MH and HD domains. This 15 amino acids stretch is 100% conserved among Diptera from *Drosophila* species to Tephritidae (*Ceratitis capitate*, *Bactrocera latifrons*, *Zeugodacus cucurbitae*), and differs by one residue (GKFLSSLNPTELTYD) in Muscidae (*Musca domestica*, *Stomoxys calcitrans*, *Lucilia cuprina*). There is no homology outside of these groups, consistent with our finding that the 48 bp FL-Ex8 is a newly evolved feature in these groups. Such strong conservation of the 15 amino acids stretch suggests that it may be functional. The strong conservation of the 3/48 bp micro-Ex8/FL-Ex8 also suggest that this alternative splicing is important, perhaps keeping a balance of the level of the two isoforms.

### Functional diversity of Hth isoforms by alternative splicing

We tested the functions of isoform Hth-A and Hth-C in vivo by (1) expressing specific isoforms in transgenic flies, (2) specific knockdown of different isoforms and (3) knockdown specific isoform and determine whether the expression of another isoform can rescue the phenotypes. In the ectopic expression experiments, Hth-A and Hth-C caused similar phenotypes in multiple tissues when ectopically expressed out of *hth* endogenous expression domain. However, when overexpressed in the *hth* endogenous expression domain, Hth-A (micro-Ex8) caused additional phenotypes (headless and wingless) not seen in Hth-C (FL-Ex8). In knockdown experiments, knockdown of either FL-Ex8 or micro-Ex8 isoforms caused pupal lethality, suggesting that the two isoforms are both required, so must have non-redundant functions. Also, the micro-Ex8 isoform, but not FL-EX8, is required for tracheal development. In the rescue experiments, Hth-C (FL-Ex8) can functionally substitute for the loss of Hth-A (micro-Ex8), but not vice versa. Altogether these results suggest a functional difference between isoforms with FL-Ex8 or micro-Ex8. They are both essential for *Drosophila* development. This is consistent with the absolute conservation of the 3/48 overlapping alternative exons organization in insects. Since micro-Ex8 is the ancient form, the newer FL-Ex8 has lost certain functions, so it cannot functionally substitute for the loss of micro-Ex8 (for tracheal development and in overexpression). FL-Ex8 must have acquired new and unique functions, so that it is also required for pupa survival.

Both Hth and Exd contain HD of the TALE (Three Amino acids Loop Extension) classes^34,59^. Hth belongs to the MEIS subclass that includes the Meis1, Meis2, Meis3, Prep1, and Prep2 in mammals and *unc-62* and *psa-2*, in the nematode *C. elegan*s. Exd belongs to the PBC subclass that includes the mammalian Pbx1, Pbx2, Pbx3, Pbx4 genes in mammal and *ceh-20, ceh-40, ceh-60* in *C. elegans*. MEIS proteins can heterodimerize with PBC proteins, heterodimerize with Hox proteins, and can also form MEIS/PBC/Hox trimeric complex^32,34,60^. The MEIS proteins can function with Hox proteins and also have Hox-independent functions^33,43,61–64^. The functional diversity may partly rely on the combination of interactions with diverse protein partners through different regions. The 15 amino acid stretch produced by FL-Ex8 lies in between the MH and HD domains. Although X-ray structure of MEIS proteins has not been reported, the 15 residue difference between micro-Ex8 and FL-Ex8 may potentially affect either the Hth protein structure or its interaction with Exd or other proteins. Therefore, the functional difference between the micro-Ex8 and FL-Ex8 isoforms may stem from differential protein interactions through this region. The alternatively spliced micro-Ex8 and FL-Ex8 exons adds to the structural diversity and potential diversity in protein interactions, providing additional layer of regulation.

### Connection with cancers

The mammalian MEIS and PBC genes are involved in the formation of various cancers^65^. Several HOX genes are also known to be involved in carcinogenesis^66^. Drugs aimed at affecting the HOX/PBX dimerization are being developed^65^. Therefore, understanding the structural and functional diversity of the MEIS, PBC and Hox proteins may provide drug targets for specific manipulation of specific protein interactions. Since each of these genes does not produce a single protein species, understanding their alternative splicing isoforms is crucial to understand their full spectrum of structural and functional diversity.

## Material and mmethods

### *Drosophila* genetics

Fly culture and crosses were performed according to standard procedure at 25 °C unless otherwise noted. Clonal analysis was generated by FLP/FRT-mediated mitotic recombination. Fly stocks used were *hth*[1422–4] ^27,28^,*hth*^*[H321]*^^45,46^,*hth*^*[P1-K6–1]*^^28^ and *hth-GAL4*^67^.

### Immunohistochemistry

Antibody staining for imaginal discs was as previously described^28^. Primary antibodies used were dG-20 (Santa Cruz Biotechnology, Inc) that specifically recognizes the C-terminal of Hth and anti-Hth-FL^28^. Species-matched Cy2- and Cy3-conjugated secondary antibodies were from Jackson ImmunoResearch. Confocal imaging was performed using Zeiss LSM microscopy.

### *hth* miRNA generation

The sequences of *hth* isoforms were obtained from FlyBase2020_01. Targeting sequences for miR-A: 5′-atacatcagaccctgatggacg; miR-C: 5′-tcggtccttcagaactaacata; The miRNA construct was generated as described in Yao et al., 2008^68^, using the method of Chen et al., 2007^69^.

### RNA-sequencing data analysis

The RNA-seq datasets were obtained from the modENCODE project GSE28078^70^ and ERP119517. The sequencing reads from the National Center for Biotechnology Information short read archive (https://www.ncbi.nlm.nih.gov/sra) were aligned to the *Drosophila melanogaster* genome version Release 6 + ISO1 MT/dm6 using STAR 2.6.1a with Ensembl reference^71^. Sequence reads were aggregated into a count for *hth* gene using htseq-count. Then read count values were transformed to RPKM with the following formula: RPKM = total exon reads/mapped reads (millions)*exon length (KB). Total reads were calculated with Flagst tools.

## Supplementary information

Supplementary Figure Legends.

Supplementary Figures.
