# Supplementary material for: Isoform-specific functions of an evolutionarily conserved 3 bp micro-exon alternatively spliced from another exon in Drosophila homothorax gene
Source: Sci Rep. 2020 Jul 30;10:12783. doi: 10.1038/s41598-020-69644-1 (PMC7392893; doi:10.1038/s41598-020-69644-1)
Supplement: Supplementary file 1 — Supplementary Figure Legends. [file 41598_2020_69644_MOESM1_ESM.docx]

**Supplementary Figure 1. Expression of FL-Ex8 and micro-Ex8 in different developmental stages**

Lane 1-3: PCR of *hth* cDNA constructs. Lane 1: cDNA clone #7 (Pai et al., 1998) representing FL-Ex8. Lanes 2, 3 and 16: cDNA clone #5 (Pai et al., 1998) representing micro-Ex8. Lanes 4-15: RT-PCR analysis of RNA from different developmental stages. Lane 4: 0-12 embryos. Lane 5: 12-18 hr embryos. Lane 6: 18-24 hr embryos. Lane 7: first instar larvae. Lane 8: second instar larvae. Lane 9: early third instar larvae. Lanes 10: mid-third instar larvae. Lane 11: late third instar larvae. Lane 12: early pupae. Lane 13: mid-pupae. Lane 14: late pupae. Lane 15: adults. The primers are 5’_21: cgctggtagtactcccggtcc, 5’_18: tggtagtactcccggtcc, 3’: cgtctgcattgcgagcat. Lanes 1 and 3 used the 5’_21 primer and produced a 164 bp and a 119 bp product, respectively. These differ by 45 bp, reflecting the length difference between FL-Ex8 and micro-Ex8. RT-PCR in lanes 4-15 detected both 164 bp and 119 bp products, indicating that both FL-Ex8 and micro-Ex8 are expressed. Lanes 2 used the 5’_18 primer, which is 18 nt, and produced a 116 bp product. Lane 16 used both 5’ primers, which differ by 3 nt, and produced products of 119 bp and 116 bp from clone #5. This demonstrated the gel is sufficient to resolve the 3 bp length difference. The fragments were separated on a 12% polyacrylamide gel. RT-PCR results showed that both FL-Ex8 and micro-Ex8 isoforms were expressed in roughly equal amount from early embryo to mid pupa (lane 4-13). The FL-Ex8 level decreased in late pupa and adult (lane 14, 15).

**Supplementary Figure 2. The relative expression of the *hth* isoforms.**

Replicates of the raw data from the modENCODE project GSE28078^65^ and ERP119517 have been analyzed. The height of the bars represents number of reads (mean ± SEM), instead of RPKM in Fig. 2. Note that HDless isoforms are more abundant in adult males and stage 16 embryos. Total reads for adult males are 13810983, 40608907; adult females are 13350053, 40756341; hemocytes from stage 16 embryo are 5925722, 6814877 and 7086806.

**Supplementary Figure 3. *Drosophila* does not have length polymorphism around exons 7 and 9**

(a) PCR primers were designed to amplify the junctions of exon 7/intron 7 and of intron 8/ exon 9. The products are 596 bp and 490 bp, respectively. (b) PCR was performed on the genomic DNA from the six homozygous viable mutant strains. In each strain, a single band was detected in the expected size for both PCR reactions.

**Supplementary Figure 4. Evolutionary conservation of the *hth* exon 8.** The Hth protein sequence coded by exon 8 was analyzed from diverse insect species: Dmel-A: *Drosophila melanogaster* isoform Hth-A; Dmel-C: *Drosophila melanogaster* isoform Hth-A; Bac: melon fly *Bactrocera latifrons* (Diptera: Tephritidae); Cer: medfly *Ceratitis capitate* (Diptera: Tephritidae); Musca: housefly *Musca domestica* (Diptera: Muscidae); Aedes: tiger mosquito *Aedes albopictus* (Diptera: [Culicidae](https://en.wikipedia.org/wiki/Culicidae)); Bombyx: silkworm *Bombyx mori* (‎Diptera: [Bombycidae](https://en.wikipedia.org/wiki/Bombycidae)); Danaus: monarch butterfly *Danaus plexippus* (‎[Lepidoptera](https://en.wikipedia.org/wiki/Lepidoptera): [Nymphalidae](https://en.wikipedia.org/wiki/Nymphalidae)); Rho: corn aphid *Rhopalosiphum maidis*([Homoptera](https://en.wikipedia.org/wiki/Homoptera): [Aphididae](https://en.wikipedia.org/wiki/Aphididae)); Acyrtho: pea aphid *Acyrthosiphon pisum* ([Homoptera](https://en.wikipedia.org/wiki/Homoptera): [Aphididae](https://en.wikipedia.org/wiki/Aphididae)); Myzus: green pea aphid *Myzus persicae* ([Homoptera](https://en.wikipedia.org/wiki/Homoptera): [Aphididae](https://en.wikipedia.org/wiki/Aphididae)). 3+48 denote the existence of the 3 bp micro-Ex8 sequence within the 48 bp FL-Ex8. 6 and 9 denote the 6 bp and 9 bp micro-Ex8.
