# Supplementary material for: Isoform-specific functions of an evolutionarily conserved 3 bp micro-exon alternatively spliced from another exon in Drosophila homothorax gene
Source: Sci Rep. 2020 Jul 30;10:12783. doi: 10.1038/s41598-020-69644-1 (PMC7392893; doi:10.1038/s41598-020-69644-1)
Supplement: Supplementary file 2 — Supplementary Figures. [file 41598_2020_69644_MOESM2_ESM.pdf]

Isoform-specific functions of an evolutionarily conserved 3 bp micro-exon alternatively spliced from another exon in *Drosophila homothorax* gene

Lin-Wen Chang[1,#], I-Chieh Tseng[1,2,#,%], Lan-Hsin Wang[3,]* and Y. Henry Sun[1,2,]*
[1]Institute of Molecular Biology, Academia Sinica, Taipei, Taiwan, Republic of China
[2]Institute of Genomic Sciences, National Yang-Ming University, Taipei, Taiwan, Republic of China
[3]Graduate Institute of Life Sciences, National Defense Medical Center, Taipei, Taiwan
*Corresponding author: lanhsinwang.sinica@gmail.com (LHW); mbyhsun@gate.sinica.edu.tw (YHS)
#These authors contributed equally to this work
[%]Current address: Department of Life Science, Chinese Culture University, Taipei, Taiwan, Republic of China

Supplementary Fig. 1

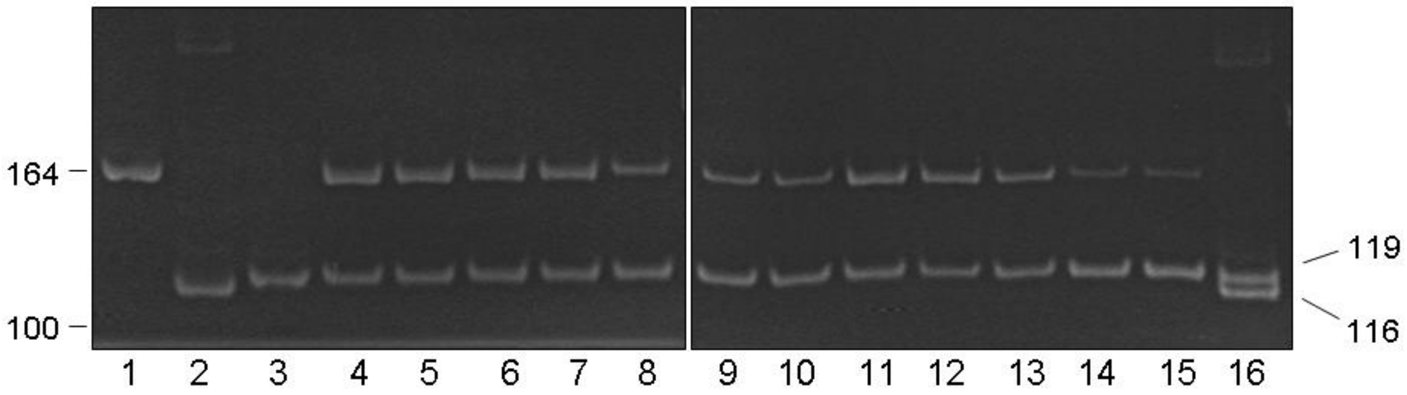

Supplementary Fig. 2

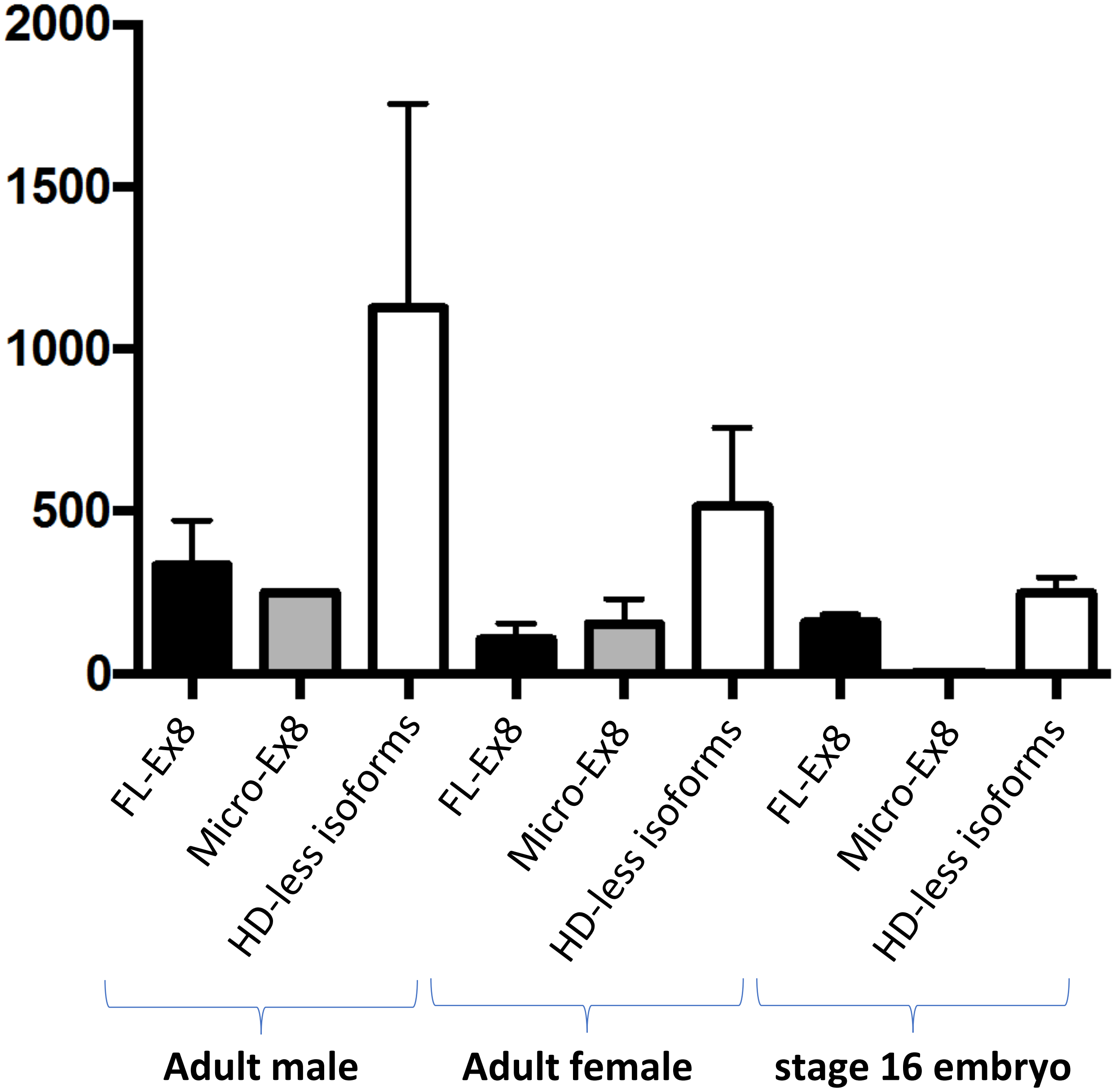

Supplementary Fig. 3

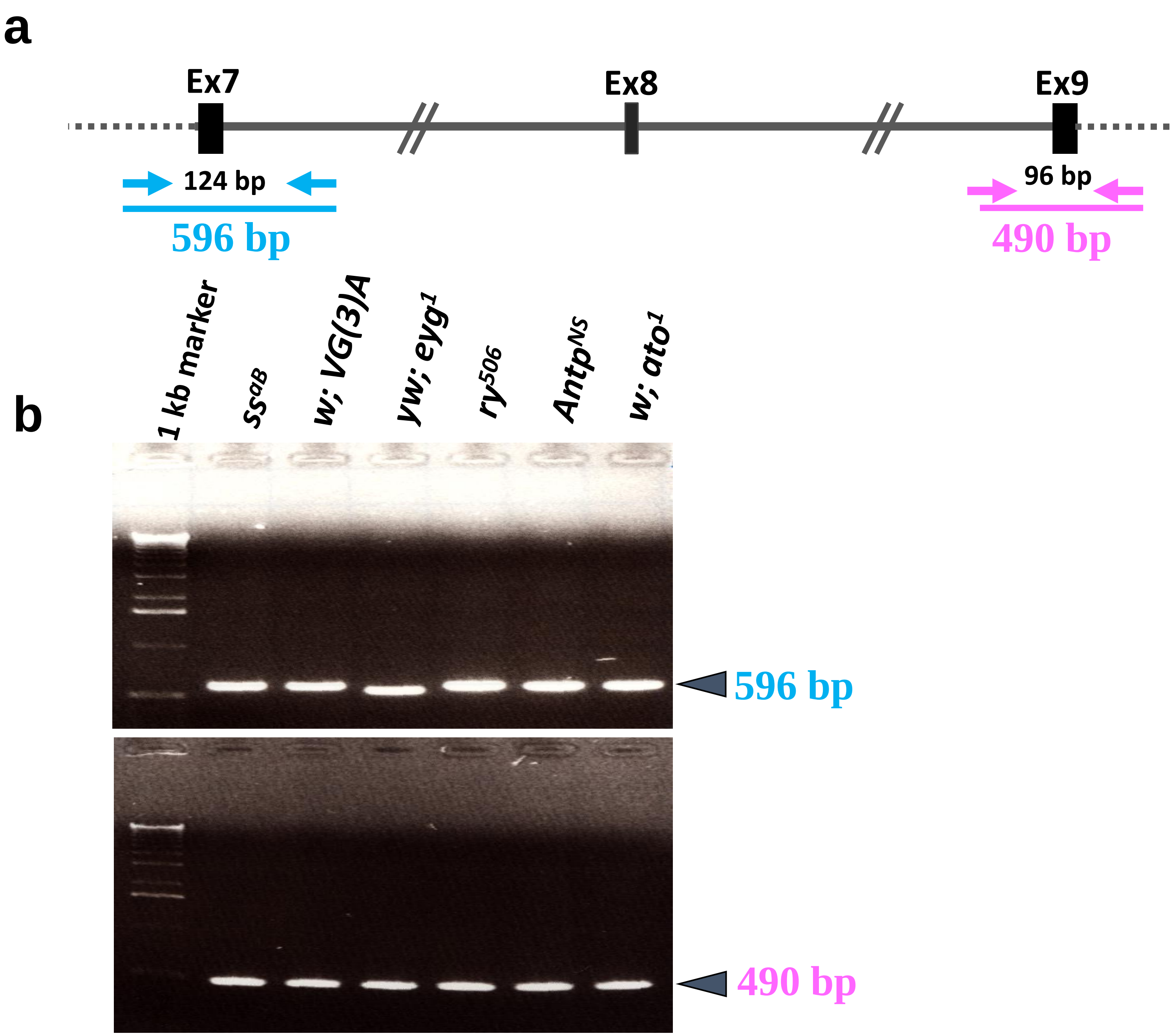

Supplementary Fig. 4

```
3     Dmel-A    NDDARSPGAGSTPGPLSQQPP-A--LDTSDPD---------------GRWCR--REWSSP
48    Dmel-C    NDDARSPGAGSTPGPLSQQPP-A--LDTSDPDGKFLSSLNPSELTYDGRWCR--REWSSP
      Bac       NDDARSPGAGSTPGPLSQQPP-V--LDTSDPDGKFLSSLNPSELTYDGRWCR--REWSSP
3+48  Cer       NDDARSPGAGSTPGPLSQQPP-V--LDTSDPDGKFLSSLNPSELTYDGRWCR--REWSSP
      Musca     NEDARSPGAGSTPGPLSQQPP-V--LDTSDPDGKFLSSLNPTELTYDGRWCR--REWSSP
3     Aedes     NDDVRSPGSGSTPGPLSAQPPPG--LDTPDPD---------------GRWCS-RRDWSSP
9     Bombyx    NDDVRSPGSGGTPGPLSQPPPQT--LDATDPDA-------------MGKWCPSRREWSSP
      Danaus    NDDVRSPGSGGTPGPLSQPPPQT--LDP-DADA-------------MGKWCGSRREWSSP
      Rho       ND-DRSPGSGGTPGPMSQQPGSQQSLDHGDPE--------------LGKWCQ-RRDWPPP
6     Acyrtho   ND-DRSPGSGGTPGPMSQQPGSQQSLDHGDPE--------------LGKWCQ-RRDWPPP
      Myzus     ND-DRSPGSGGTPGPMSQQPGSQQSLDHGDPE--------------LGKWCQ-RRDWPPP
                *:  ****:*.****:*  *     **   *                *:**   *:*  *
```

Supplementary Figure 1. Expression of FL-Ex8 and micro-Ex8 in different developmental stages

Lane 1-3: PCR of *hth* cDNA constructs. Lane 1: cDNA clone #7 (Pai et al., 1998) representing FL-Ex8. Lanes 2, 3 and 16: cDNA clone #5 (Pai et al., 1998) representing micro-Ex8. Lanes 4-15: RT-PCR analysis of RNA from different developmental stages. Lane 4: 0-12 embryos. Lane 5: 12-18 hr embryos. Lane 6: 18-24 hr embryos. Lane 7: first instar larvae. Lane 8: second instar larvae. Lane 9: early third instar larvae. Lanes 10: mid-third instar larvae. Lane 11: late third instar larvae. Lane 12: early pupae. Lane 13: mid-pupae. Lane 14: late pupae. Lane 15: adults. The primers are 5'_21: cgctggtagtactcccggtcc, 5'_18: tggtagtactcccggtcc, 3': cgtctgcattgcgagcat. Lanes 1 and 3 used the 5'_21 primer and produced a 164 bp and a 119 bp product, respectively. These differ by 45 bp, reflecting the length difference between FL-Ex8 and micro-Ex8. RT-PCR in lanes 4-15 detected both 164 bp and 119 bp products, indicating that both FL-Ex8 and micro-Ex8 are expressed. Lanes 2 used the 5'_18 primer, which is 18 nt, and produced a 116 bp product. Lane 16 used both 5' primers, which differ by 3 nt, and produced products of 119 bp and 116 bp from clone #5. This demonstrated the gel is sufficient to resolve the 3 bp length difference. The fragments were separated on a 12% polyacrylamide gel. RT-PCR results showed that both FL-Ex8 and micro-Ex8 isoforms were expressed in roughly equal amount from early embryo to mid pupa (lane 4-13). The FL-Ex8 level decreased in late pupa and adult (lane 14, 15).

Supplementary Figure 2. The relative expression of the *hth* isoforms.

Replicates of the raw data from the modENCODE project GSE28078[72] and ERP119517 have been analyzed. The height of the bars represents number of reads (mean ± SEM), instead of RPKM in Fig. 2. Note that HDless isoforms are more abundant in adult males and stage 16 embryos. Total reads for adult males are 13810983, 40608907; adult females are 13350053, 40756341; hemocytes from stage 16 embryo are 5925722, 6814877 and 7086806.

Supplementary Figure 3. *Drosophila* does not have length polymorphism around exons 7 and 9

(a) PCR primers were designed to amplify the junctions of exon 7/intron 7 and of intron 8/ exon 9. The products are 596 bp and 490 bp, respectively. (b) PCR was performed on the genomic DNA from the six homozygous viable mutant strains. In each strain, a single band was detected in the expected size for both PCR reactions.

Supplementary Figure 4. Evolutionary conservation of the *hth* exon 8. The Hth protein sequence coded by exon 8 was analyzed from diverse insect species: Dmel-A: *Drosophila melanogaster* isoform Hth-A; Dmel-C: *Drosophila melanogaster* isoform Hth-A; Bac: melon fly *Bactrocera latifrons* (Diptera: Tephritidae); Cer: medfly *Ceratitis capitate* (Diptera: Tephritidae); Musca: housefly *Musca domestica* (Diptera: Muscidae); Aedes: tiger mosquito *Aedes albopictus* (Diptera: Culicidae); Bombyx: silkworm *Bombyx mori* (Diptera: Bombycidae); Danaus: monarch butterfly *Danaus plexippus* (Lepidoptera: Nymphalidae); Rho: corn aphid *Rhopalosiphum maidis* (Homoptera: Aphididae); Acyrtho: pea aphid *Acyrthosiphon pisum* (Homoptera: Aphididae); Myzus: green pea aphid *Myzus persicae* (Homoptera: Aphididae). 3+48 denote the existence of the 3 bp micro-Ex8 sequence within the 48 bp FL-Ex8. 6 and 9 denote the 6 bp and 9 bp micro-Ex8.

Table 1. The specific 5' splice site rule for the *hth* genes in insects

The 3' and 5' splice recognition sites for the internal exons in the *hth* gene from diverse insect species: *Drosophila melanogaster* (Diptera: Drosophilidae), *Aedes aegypti* (Diptera: Culicidae), *Anopheles gambiae* (Diptera: Culicidae), *Bombyx mori* (Lepidoptera), *Tribolium castaneum* (Coleoptera), *Apis mellifera* (Hymenoptera) and *Acyrthosiphon pisum* (Hemiptera). The length of each exon is also listed. The exon boundary of the internal exons in *hth* can be defined by CAG-exon-GTGAGT or CAG-exon-GTAAGT, which is a specific subset of the 5' splice site motifs (Yeo and Burge, 2004). The length of exons 3, 4, 5, 12 and 13 are conserved. The other exons vary slightly in length, but the variations are always in-frame. The 24 bp exon 10 of *hth* is absent in all of the tested non-*Drosophila* species.

Table 2. Phenotypes of overexpression and/or knockdown of specific isoform.

The phenotypes of overexpressing isoforms Hth-A and Hth-C and specific knockdown by miR-A and miR-C are described. The phenotypes of coexpressing different miR with Hth-A or Hth-C are also described.
